# Investigation of the inhibitory effects of total flavonoids of litchi seed on *Clonorchis sinensis*-induced liver damage and fibrosis

**DOI:** 10.3389/fcimb.2025.1625004

**Published:** 2025-07-30

**Authors:** Liuchun Luo, Jilong Wang, Qiuchen Cheng, Zuochao Lu, Jiahui Lv, Suyu Xiao, Lili Tang, Shanshan He, Dengyu Liu, Zeli Tang, Qing Li, Tingzheng Zhan

**Affiliations:** ^1^ Department of Parasitology, Guangxi Medical University, Nanning, China; ^2^ Department of Clinical Laboratory, Liuzhou People’s Hospital, Liuzhou, China; ^3^ Department of Gastroenterology, the People’s Hospital of Guangxi Zhuang Autonomous Region, Guangxi Academy of Medical Sciences, Nanning, China; ^4^ Department of Cell Biology and Genetics, Guangxi Medical University, Nanning, China; ^5^ Key Laboratory of Longevity and Aging-Related Diseases of Chinese Ministry of Education, Guangxi Medical University, Nanning, China; ^6^ Key Laboratory of Basic Research on Regional Diseases (Guangxi Medical University), Education Department of Guangxi Zhuang Autonomous Region, Nanning, China

**Keywords:** *Clonorchis sinensis*, clonorchiasis, total flavonoid of litchi seed, liver fibrosis, hepatic stellate cell

## Abstract

**Background:**

*Clonorchis sinensis* (*C. sinensis*), which is prevalent in Asian countries, including China, Korea and Vietnam, is known to cause liver fibrosis, leading to various liver diseases and potentially fatal outcomes. Total flavonoids of litchi seed (TFL), a traditional Chinese medicine abundant in the Southern China, is known for its multiple pharmacological activities, including anti-fibrotic, anti-oxidative and hepato-protective properties. The present study explored the inhibitory effects of TFL on liver damage caused by *C. sinensis* infection in rats.

**Methods:**

In the animal experiment, female rats were infected with *C. sinensis* and treated with TFL. Serum biochemical indicators were measured each week. Pathological changes in the rat liver were evaluated by examining the appearance of liver, and by performing hematoxylin and eosin (H&E) and Masson’s trichrome staining. Through immunofluorescence and immunohistochemistry, the number of activated hepatic stellate cells (HSCs) were counted and the collagen deposition area in these cells were measured. In the cell experiment, rat hepatic stellate cells (HSC-T6) were stimulated with TGF-β1 (10 ng/ml) and treated with different concentrations of TFL. The expression levels of HSCs activation markers α-smooth muscle actin (α-SMA), collagen I, and fibronectin were detected using quantitative real-time polymerase chain reaction (RT-qPCR). The protein levels of α-SMA and collagen I was detected by Western blot.

**Results:**

TFL improved liver function in *C. sinensis*-infected rats, as indicated by reduced levels of transaminases, bilirubin, and bile acids. TFL also alleviated pathological changes in liver tissues. TFL also reduced the number of activated HSCs and downregulated fibrosis-related markers (collagen I/III, α-SMA) in rat liver. In HSC-T6, TFL inhibited HSCs activation by reducing the mRNA levels of α-SMA, collagen I, and fibronectin, and reduced the protein level of α-SMA and collagen I.

**Conclusions:**

TFL attenuated *C. sinensis*-induced liver fibrosis in rats. Our study provided experimental evidence for the development of novel anti-liver fibrosis drugs and offer new insights into the treatment of *C. sinensis* infections.

## 1 Introduction

*C. sinensis*, a significant but under-recognized foodborne parasite, is endemic in parts of Asia, particularly China, Korea, and Vietnam. In China, infections cluster in southern (Guangxi, Guangdong) and northeastern (Heilongjiang) regions. Guangxi’s high infection rates are linked to traditional raw fish consumption. Recent data indicate rising prevalence in some areas, with expanding geographic distribution and a notable burden of moderate-to-severe cases. Consequently, *C. sinensis* is now a priority for parasitic disease control in China (Lai et al., 2017; Jiang et al., 2021; Xie et al., 2022).

*C. sinensis* infection manifests variably, from asymptomatic/mild cases to severe hepatobiliary pathologies. While light infestations often lack overt symptoms, heavy burdens can cause liver fibrosis progressing to cirrhosis (Na et al., 2020), alongside fever, jaundice, cholangitis, and cholangiocarcinoma. Notably, this WHO-classified neglected tropical disease elevates liver cancer risk (Squadroni et al., 2017; O’Rourke, 2024) and endangers ~200 million people globally (Hong and Fang, 2012).

Litchi seed, the dried mature seed of *Litchi chinensis*, contains flavonoids and saponins as its primary bioactive components (Liu et al., 2023). Total flavonoids of litchi seed (TFL), a traditional Chinese medicine, exhibit hepatoprotective, anti-fibrotic, hypoglycemic, and hypolipidemic effects, along with antioxidant and free radical-scavenging properties (Zhu et al., 2019). TFL has demonstrated anti-fibrotic efficacy in both cholestatic and carbon tetrachloride (CCl_4_)-induced liver fibrosis models (Yan et al., 2022). Notably, litchi seed extracts (alcohol: 182 g/kg; water: 87 g/kg) show no toxicity in mice even at high gavage doses (Wan et al., 2010). However, its potential therapeutic effects on *C. sinensis*-induced liver injury remain unexplored.

This study evaluated the inhibitory effects of TFL on *C. sinensis*-induced liver damage using both rat models and *in vitro* cell experiments. The findings will provide experimental evidence for developing novel natural anti-fibrotic drugs and contribute new therapeutic perspectives for *C. sinensis* infection.

## 2 Materials and methods

### 2.1 Ethics statement

All animal procedures were authorized by the Institutional Animal Care and Use Committee (IACUC) of Guangxi Medical University for the use of laboratory animals (approval no. 202309001 and 202402003).

### 2.2 Preparation of TFL

TFL was purchased from Nanjing Herb Source Biotechnology Co., Ltd. (Nanjing, China) and the chemical components of TFL were identified by Tsinghua University (Beijing, China). Detailed chemical components, extraction and purification methods of TFL can be found in references (Tang, 2007; Yu, 2019; Qian et al., 2020; Yan et al., 2022).

### 2.3 Preparation of metacercaria

*C. sinensis* metacercariae were isolated from infected *Pseudorasbora parva*. Fish were euthanized by 30 min immersion in 400 mg/L MS-222 (confirmed by cessation of respiration and rhythmic eye movements). After removing heads, scales, viscera and bones, muscle tissue was weighed and homogenized. Artificial digestion solution (10 ml HCl + 6 g gastric protease in 1000 ml saline) was added (1:10 w/v ratio). The mixture was incubated at 37°C overnight, filtered (80-mesh sieve), and the filtrate settled in a 2000 ml flask for 40 min. After three PBS wash cycles, sediment was transferred to a glass dish. Metacercariae were isolated microscopically and stored in 1.5 ml tubes (Jiang et al., 2024).

### 2.4 Animal experiment and tissue collection

**Figure 1:**
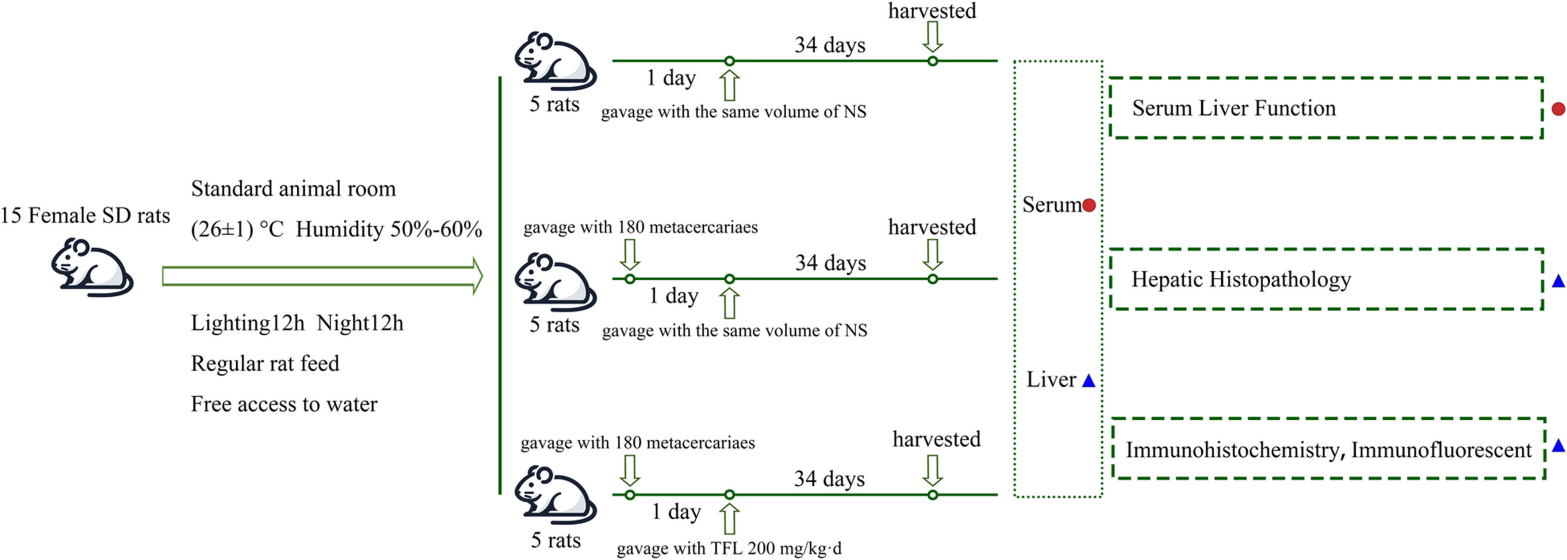
Diagram of rat grouping and model construction.

Fifteen healthy female rats were randomly divided into control, *C. sinensis* infection (*Cs*), and TFL treatment groups (*Cs*+TFL), with five rats in each group. Animals were housed under standard conditions (26 ± 1°C, 50-60% humidity, 12 h light/dark cycle) with free access to food and water. Infection was established by oral gavage of 180 metacercariae per rat (excluding controls). TFL was dissolved in pure water to prepare solutions. From infection day 1, the *Cs*+TFL group received daily 200 mg/kg TFL by oral gavage while other groups received water only. Consistent with previous findings that experimental animals had the most severe lesions at day 35 (5th week) post-infection (Zhan et al., 2023), weekly tail vein blood samples were collected using hypodermic needles. On day 35, rats were euthanized by CO_2_ asphyxia (30% volume displacement/min), confirmed by 5 min observation of vital signs cessation. Liver and serum samples were collected post-dissection (**Figure 1**). The adult worms were collected from bile duct by saline flushing, and the worm number was counted.

### 2.5 Serum biochemical analysis

A fully automated biochemical analyzer and its corresponding reagents (Roche Diagnostics GmbH) were used to measure liver function indicators in rat serum, including alanine aminotransferase (ALT), aspartate aminotransferase (AST), total bile acids (TBA) and alkaline phosphatase (ALP), to assess the extent of liver damage in rats. Enzyme levels and TBA are expressed in units per liter (U/L) and micromoles per liter (μmol/L), respectively.

### 2.6 Histopathological, immunohistochemical and immunofluorescent analysis

Liver tissues were fixed in 4% paraformaldehyde at 4°C for 48 h and subsequently paraffin-embedded. Tissue sections (4-µm thick) were prepared and stained with hematoxylin (4 min) and eosin (2 min) (H&E; cat. no. G1120; Solarbio; China), followed by Masson’s trichrome staining (25 min at room temperature; cat. no. G1340; Solarbio; China) according to the manufacturer’s instructions. Following staining, sections were deparaffinized in xylene and rehydrated through an ethanol gradient series at room temperature. Antigen retrieval was performed by microwave heating in citrate buffer (15 min), followed by PBS washes. Endogenous peroxidase activity was blocked with 3% H_2_O_2_ (10 min at room temperature; cat. no. P0100B; Beyotime Biotechnology; China), followed by blocking with 10% goat serum (10 min at room temperature; cat. no. ZLI-9022; Zhongshan Golden Bridge Biotechnology; China).

For immunohistochemistry, sections were incubated overnight at 4°C with the following primary antibodies (all from Servicebio, China): rabbit anti-α-SMA (1:300; cat. no. GB111364-100), rabbit anti-collagen I (1:300; cat. no. GB11022-3-100), and rabbit anti-collagen III (1:300; cat. no. GB111629-100). After PBS washes, sections were incubated with HRP-conjugated goat anti-rabbit IgG secondary antibody (1:300, 1 h at 37°C; cat. no. GB23303; Servicebio; China), developed with 0.05% DAB for 80 s, and counterstained with hematoxylin (4 min at room temperature). Finally, sections were dehydrated through an ethanol gradient and mounted with neutral balsam.

For immunofluorescence, sections were incubated with rabbit anti-desmin (1:500; cat. no. GB15075-100; Servicebio; China) followed by Alexa Fluor 594-conjugated goat anti-rabbit IgG (1:500; cat. no. ab150077; Abcam; UK). Nuclei were counterstained with DAPI (1 µg/ml, 15 min in the dark at room temperature). Three randomly selected fields per slide were imaged using a Leica fluorescence microscope (Germany). The positive staining area to total tissue area ratio was quantified using ImageJ software (v1.0; NIH, USA), with the mean of three measurements used for statistical analysis.

### 2.7 Cell culture and RT-qPCR

Previous studies indicated that liver fibrosis was closely associated with the activation of HSCs. In cell experiment, TGF-β1 was used to activate HSCs. Initially, rat HSC-T6 were cultured in high-glucose DMEM medium (cat. no. SA112.02; CellMax; China) supplemented with 10% FBS (cat. no. CGM101.05; CellMax; China) and incubated in an incubator at 37°C with 5% CO_2_, with passages every 3–4 days. Cell viability under different TFL concentrations was assessed using the CCK-8 assay (cat. no. MA0218-5; Meilunbio; China). To study TFL’s effect on HSCs activation, log-phase HSC-T6 cells were seeded in 6-well plates (1×10^5^ cells/well). When cells reached 60-70% confluence (Wolfe et al., 2008), the seeded T6 cells were divided into four groups: Control, TGF-β1 (10 ng/ml), TGF-β1 + 150 mg/l TFL(TFL-Low), and TGF-β1 + 450 mg/l TFL (TFL-High). Both TGF-β1 and TFL were dissolved in medium containing 10% FBS. Cells were harvested 48 h after TGF-β1 and TFL treatments. Total RNA was extracted from cells using the Cell Total RNA Isolation Kit (cat. no. RE-03113; Foregene; China), followed by cDNA synthesis with the Reverse Transcription Kit (cat. no. TAU341-02; TransGen; China) according to manufacturer’s protocols. RT-qPCR primer sequences are listed in **Table 1**.

**Table 1:** RT-qPCR primer sequences.

Primers	Sequences
GAPDH forward	5’-ATGGGAAGCTGGTCATCAAC-3’
GAPDH reverse	5’- GTGGTTCACACCCATCACAA-3’
α-SMA forward	5’- GGACGTACAACTGGTATTGTG-3
α-SMA reverse	5’- TCAGCAGTAGTCACGAAGGAAT-3
Collagen I forward	5’-TCGGCGAGAGCATGACCGATGGAT-3
Collagen I reverse	5’-GACGCTGTAGGTGAAGCGGCTGTT-3
Fibronectin forward	5’-GACCAGGTTGATGACACTTCCATTG-3
Fibronectin reverse	5’- TGAGTTCTGTGCTACTGCCTTCTAC-3

GAPDH served as the endogenous control. The RT-qPCR thermocycling conditions were as follows: 95°C for 3 min, followed by 40 cycles of 95°C for 15 s and 60°C for 30 s. The reaction volume was 10 µl. Gene expression fold-changes were calculated using the 2^−ΔΔCt^ method.

### 2.8 Western blot

The protein concentration was measured using the BCA Protein Assay Kit (cat. no. ZJ101; Epizyme Biomedical Technology Co., Ltd.; China). The protein samples were separated and purified using a 12% polyacrylamide gel. Following electrophoresis, the proteins were transferred to a cellulose acetate membrane. The membrane was blocked with 5% skimmed milk at room temperature for 2 h, then incubated overnight at 4°C with anti-α-smooth muscle actin (dilution 1:1,000; cat. no. 19245; Cell Signaling Technology, Inc.; USA), anti-collagen I (dilution 1:1,000; HA722517; HuaBio, Inc.; China) and Anti-β-tubulin (dilution 1:1,000; cat. no. GB11017-100; Servicebio, Inc.; China) as the primary antibodies. Following washing with TBST, the membrane was incubated for 1 h at 37°C with anti-rabbit IgG secondary antibody (dilution 1:10,000; cat. no. 35401; Cell Signaling Technology, Inc.; USA). Following another wash with TBST, the images were captured using the Odyssey Infrared Fluorescence Scanning Imaging System (LI-COR Biosciences), and analyzed using ImageJ software.

### 2.9 Statistical analysis

All quantitative data are expressed as the mean ± standard error from three independent experiments. Experimental data were analyzed by one-way analysis of variance (ANOVA) followed by Bonferroni *post-hoc* test. Perform a Kruskal-Wallis test on non-normally distributed data, followed by a Dunn’s *post-hoc* test. All statistical graphs were generated using GraphPad Prism software (version 9.0; GraphPad Software, Inc.). Statistical analysis was performed using SPSS software (version 25.0; SPSS Inc.). *P*<0.05 was considered to indicate a statistically significant difference.

## 3 Results

### 3.1 Components of TFL

**Figure 2:**
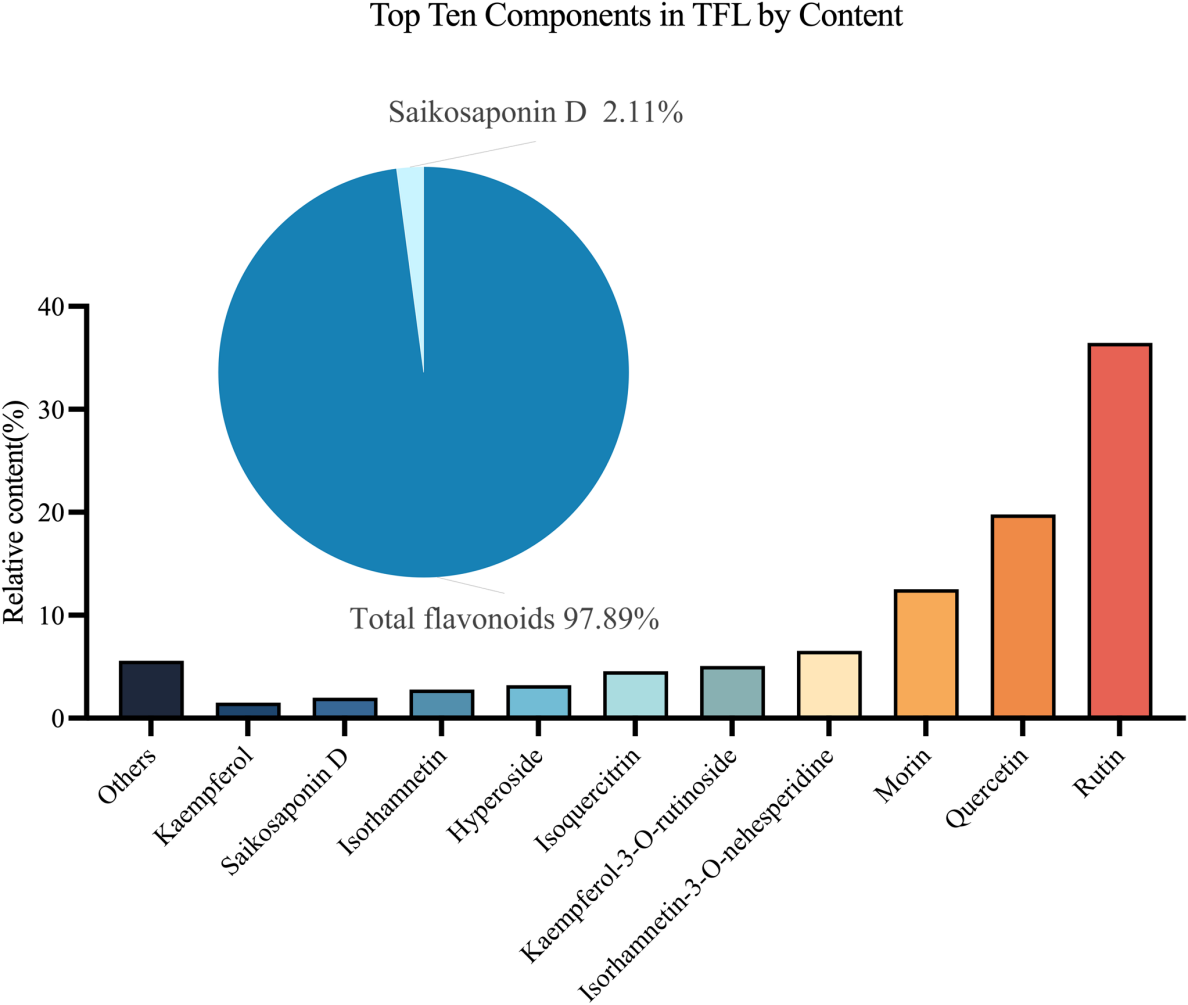
Analysis of TFL Components. The top ten compounds by content are: rutin, quercetin, morin, isorhamnetin-3-O-neohesperidoside, kaempferol-3-O-rutinoside, isoquercitrin, hyperoside, isorhamnetin, saikosaponin D, and kaempferol.

By analyzing the chemical composition of TFL, it was found to contain over 40 compounds (**Supplementary Table 1**). The top ten compounds accounted for 94.43% of all compounds, with total flavonoids making up 97.89% of these compounds. The three most abundant substances were rutin (36.4%), quercetin (19.8%), and morin (12.5%) (**Figure 2**).

### 3.2 TFL modulated liver function in *C. sinensis-*infected rats

**Figure 3:**
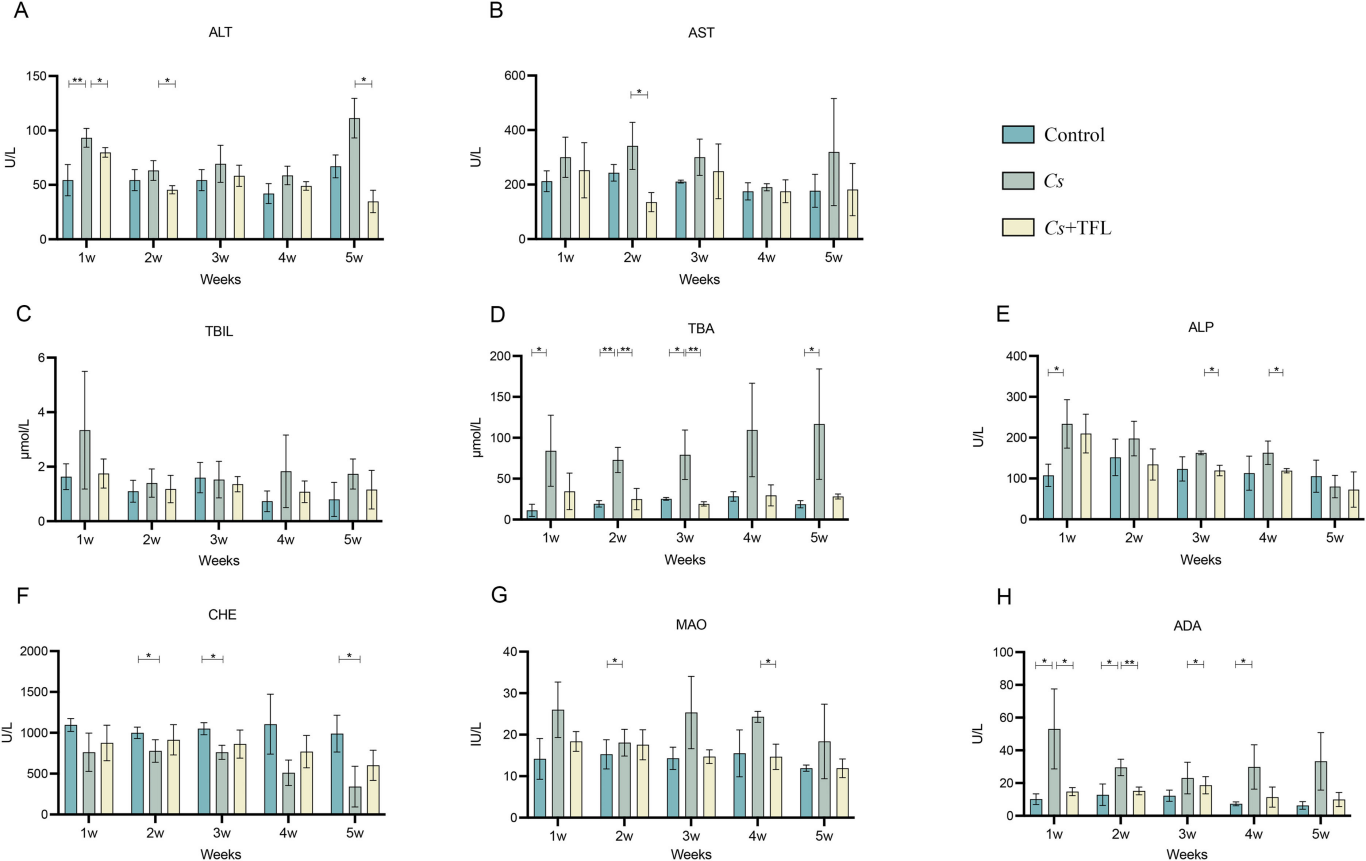
Comparison of liver function in different groups of experimental rats. **(A)** ALT, **(B)** AST, **(C)** TBIL, **(D)** TBA, **(E)** ALP, **(F)** CHE, **(G)** MAO and **(H)** ADA levels. All data were statistically analyzed using one-way ANOVA followed by Bonferroni *post-hoc* test (n=5). *P<0.05 and **P<0.01. ALT, alanine aminotransferase; AST, aspartate aminotransferase; TBIL, total bilirubin; TBA, total bile acids; ALP, alkaline phosphatase; CHE, cholinesterase; MAO, monoamine oxidase; ADA, adenosine deaminase; *Cs*, *Clonorchis sinensis*.

No significant difference was observed in *C. sinensis* adult worm burdens between the *Cs* and *Cs*+TFL groups (49 ± 19.8 *vs*. 42 ± 15.7). However, TFL treatment significantly modulated serum liver function parameters compared to the *Cs* group. Both ALT (significantly decreased) and AST (non-significantly decreased) showed reductions, though to different degrees, consistent with their roles as hepatocellular damage biomarkers. (**Figures 3A, B**). *C. sinensis* infection-induced liver dysfunction was characterized by elevated liver transaminases and systemic accumulation of bile components (TBIL, TBA, ALP). TFL treatment consistently reduced TBIL, TBA, and ALP levels versus the *Cs* group across time points, though with differential statistical significance among individual markers(**Figures 3C–E**). The levels of cholinesterase (CHE), monoamine oxidase (MAO) and adenosine deaminase (ADA) also demonstrated improvement trends. (**Figures 3F–H**).

### 3.3 TFL improved liver histopathology in *C. sinensis-*infected rats

**Figure 4:**
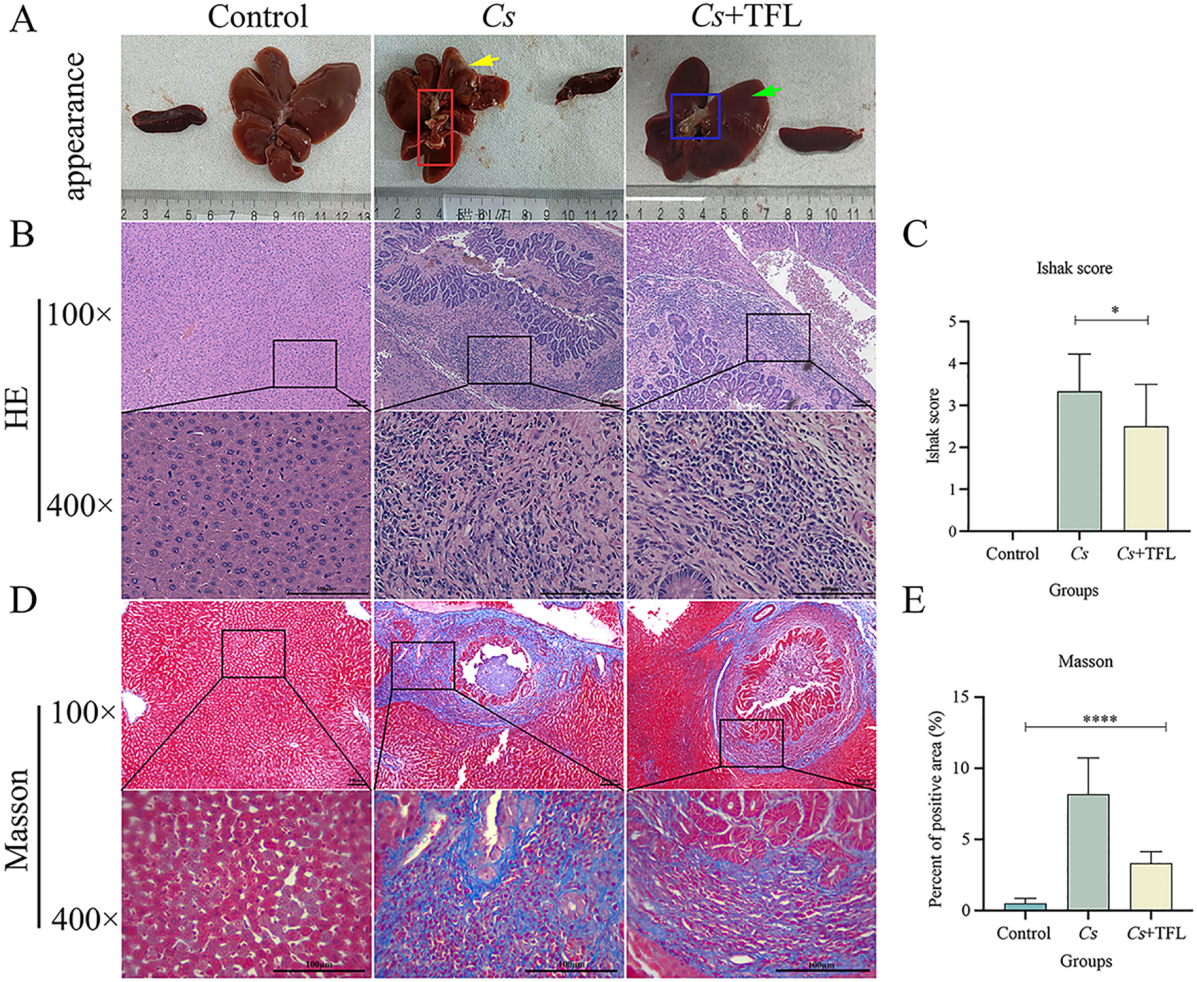
Comparison of hepatic histopathology in rats. **(A)** Appearance of rat spleen and liver. **(B)** Rat liver tissue pathological changes observed with H&E staining (scale bars, 100 μm). **(C)** Rat liver fibrosis scoring. **(D)** Observation of liver fibrosis in rats with Masson’s Trichrome staining (scale bars, 100 μm). **(E)** Comparison of the area of Masson’s Trichrome staining in rat liver. H&E staining was statistically analyzed using one-way ANOVA followed by Bonferroni *post-hoc* test. Masson’s Trichrome staining was statistically analyzed using Kruskal-Wallis test followed by Dunn’s *post-hoc* test. Data are presented as mean ± SD (n=5). *P<0.05 and ****P<0.0001.

In healthy rats, livers displayed characteristic red coloration, smooth surfaces, and natural gloss. Following *C. sinensis* infection, livers exhibited significant swelling, bile duct dilation containing numerous adult worms (red box, **Figure 4A**), and parenchymal yellowing (yellow arrow, **Figure 4A**). TFL treatment reduced hepatic swelling, diminished bile duct dilation (blue box, **Figure 4A**), lightened parenchymal coloration, and restored bile duct surface gloss (green arrow, **Figure 4A**). Histopathological assessment of H&E-stained sections (**Figure 4B**) demonstrated that control livers exhibited characteristic hepatic architecture with radially arranged hepatocytes, intact lobular structures, and well-defined portal areas, devoid of significant collagen deposition or inflammatory infiltrates. *C. sinensis*-infected livers displayed severe architectural disruption, featuring disorganized hepatocyte arrangement, fragmented lobular boundaries, obscured portal triads, dilated and congested central veins, and extensive inflammatory cell infiltration. TFL treatment markedly improved histology, with near-normal hepatocyte alignment, reconstructed lobules, clearer portal areas, reduced central vein congestion, and significantly diminished inflammation compared to the *Cs* group.

Masson’s trichrome staining also demonstrated significant fibrotic changes among experimental groups (**Figures 4C–E**). *C. sinensis*-infected rats exhibited markedly increased collagen deposition in livers (one-way ANOVA, *F*_(3,8)_=5.926, *P* = 0.002) as quantified by both collagen content measurement and Ishak fibrosis scoring. TFL treatment substantially attenuated these fibrotic alterations, showing significantly reduced collagen fiber accumulation (one-way ANOVA, *F*_(3,8)_=13.915, *P* = 0.004) and lower Ishak scores (one-way ANOVA, *F*_(3,8)_=8.915, *P* = 0.02) compared to the *Cs* group.

### 3.4 Effect of TFL on key markers in liver tissue of rats infected with *C. sinensis*

**Figure 5:**
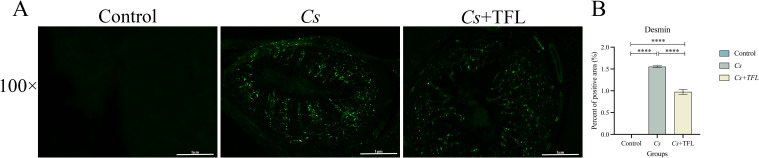
**(A)** Comparison of desmin expression levels in rat liver tissue. **(B)** Statistical comparison of desmin expression levels in rat liver tissue (scale bars, 100 μm). All data were statistically analyzed using one-way ANOVA followed by Bonferroni *post-hoc* test. Data are presented as mean ± SD (n=5). ****P<0.0001.

**Figure 6:**
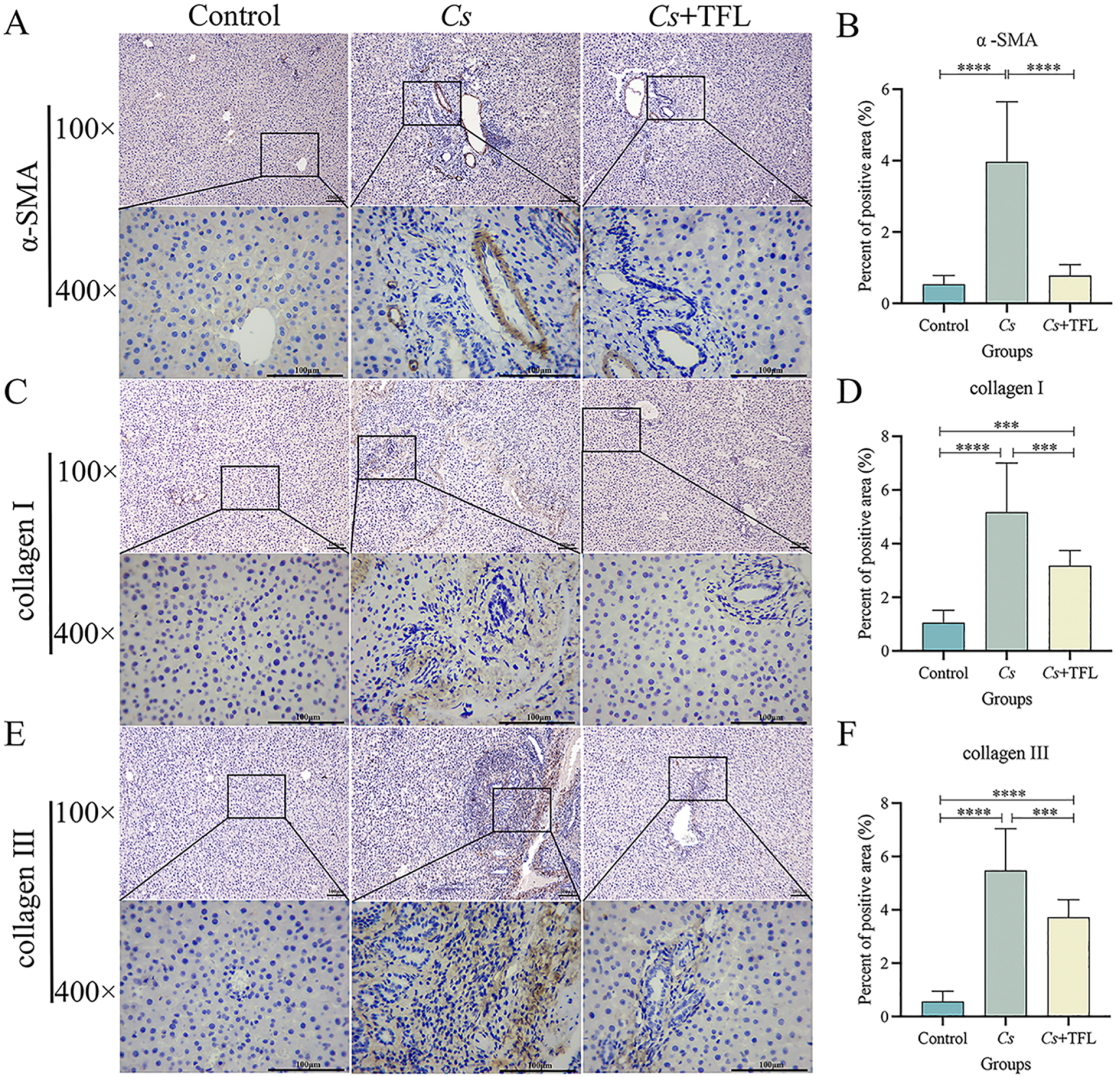
Comparison of collagen I and III, and α-SMA deposition areas in rat liver tissue. **(A)** Comparison and **(B)** statistical analysis of α-SMA deposition area in rat liver tissue (scale bars, 100 μm). **(C)** Comparison and **(D)** statistical analysis of collagen I deposition in rat liver tissue (scale bars, 100 μm). **(E)** Comparison and **(F)** statistical analysis on of collagen III deposition in rat liver tissue (scale bars, 100 μm). Collagen I and collagen III were statistically analyzed using one-way ANOVA followed by Bonferroni *post-hoc* test; α-SMA was statistically analyzed using Kruskal-Wallis test followed by Dunn’s *post-hoc* test. Data are presented as mean ± SD (n=5). ***P<0.001 and ****P<0.0001.

Desmin fluorescence and collagen deposition areas in rat livers were analyzed using ImageJ and SPSS software. Compared with the *Cs* group, desmin expression in the liver tissue of rats with TFL treatment decreased (**Figure 5**). The levels of collagen I (**Figures 6A, B**, one-way ANOVA, *F*_(3,8)_=9.459, *P* = 0.04) and collagen III (**Figures 6C, D**, one-way ANOVA, *F*_(3,8)_=8.436, *P* = 0.003), and α-SMA (**Figures 6E, F**, one-way ANOVA, *F*_(3,8)_=12.627, *P* = 0.0006) were reduced with statistically significance.

### 3.5 TFL inhibited HSCs activation

**Figure 7:**
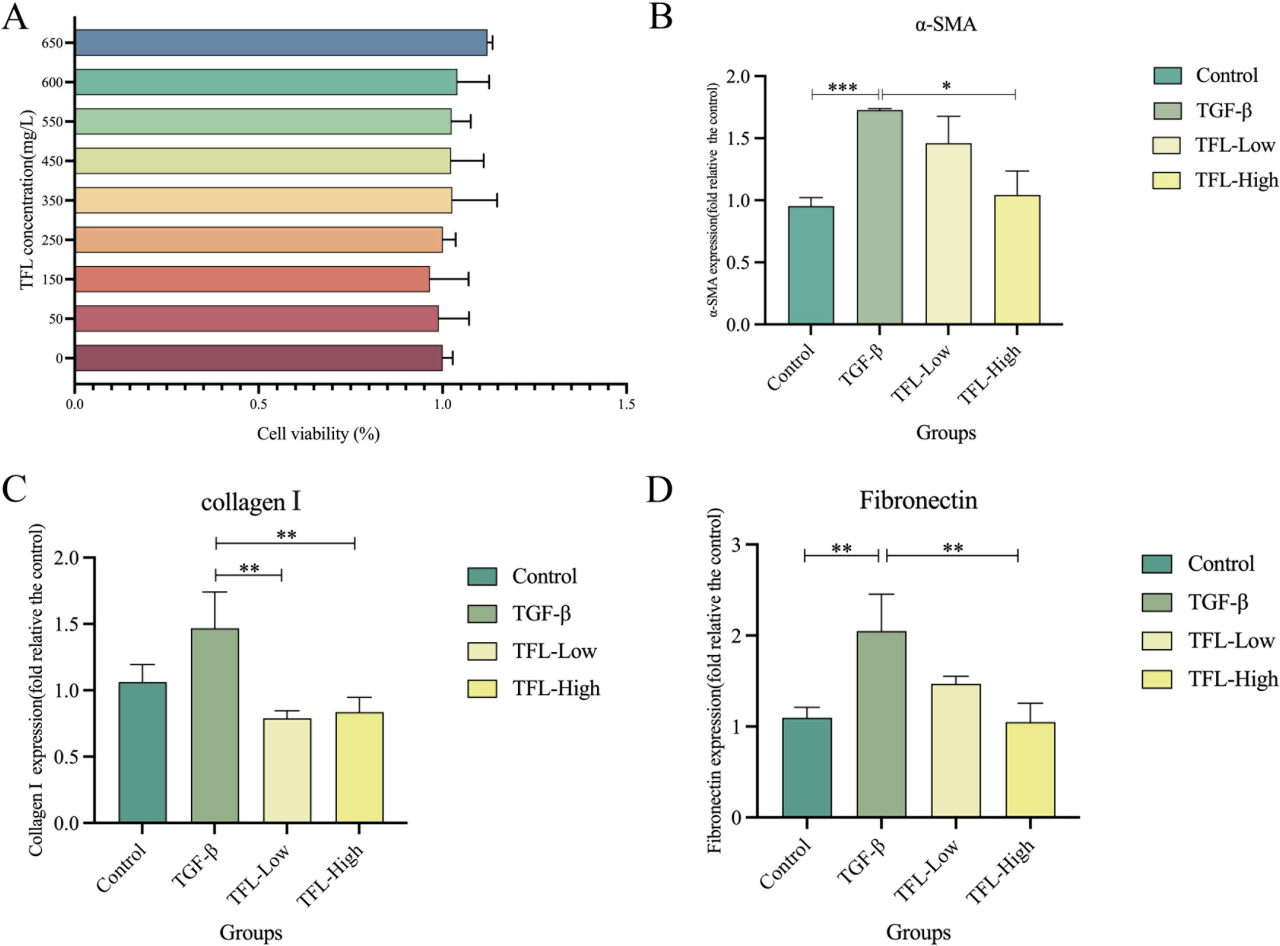
Effects of different concentrations of TFL on The gene expression in TGF-β1 activated HSC-T6 cells. **(A)** Assessment of TFL impact on HSC using CCK-8. **(B)** RT-qPCR analysis of α-SMA expression. **(C)** RT-qPCR analysis of collagen I expression. **(D)** RT-qPCR analysis of fibronectin expression. Results are expressed as normalized fold values relative to the control. All data were statistically analyzed using one-way ANOVA followed by Bonferroni *post-hoc* test. Data are presented as mean ± SD (n=5). *P<0.05, **P<0.01 and ***P<0.001.

**Figure 8:**
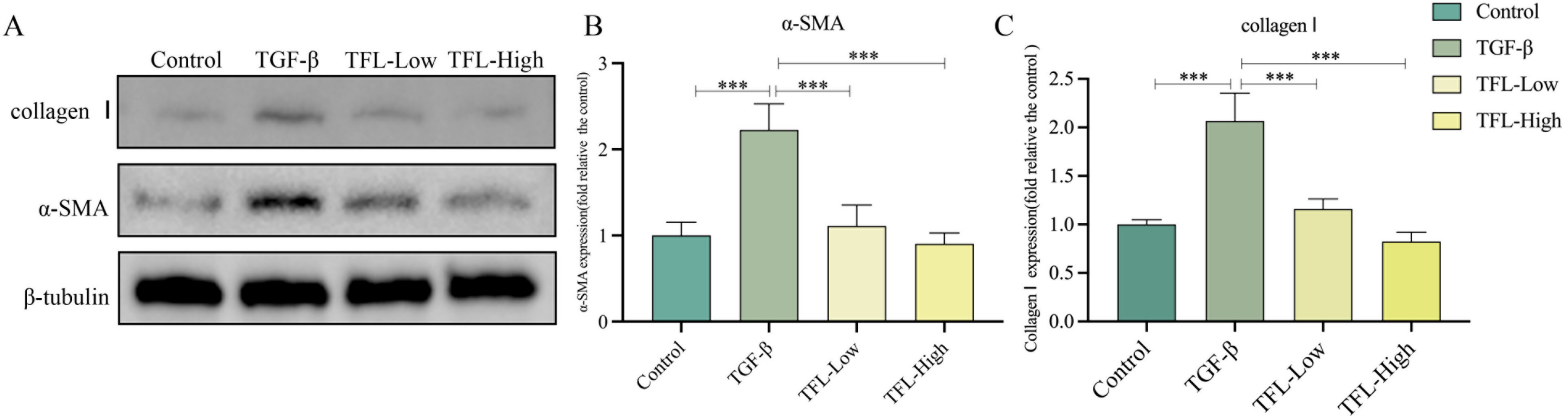
Effects of different concentrations of TFL on The protein expressions in in TGF-β1 activated HSC-T6 cells. **(A)** Western blot analysis. Lane 1, Control group; Lane 2, TGF-β1-activated group; Lane 3, TFL-Low group; Lane 4, TFL-High group. **(B, C)** Quantification of all bands in **(A)**.

The effect of TFL on HSCs viability was assessed using cell counting kit-8, demonstrating that high concentrations of TFL are non-toxic to rat HSCs (**Figure 7A**). To evaluate TFL’s antifibrotic potential, we examined its impact on HSCs activation induced by TGF-β1, a key profibrogenic cytokine in liver fibrogenesis. Following TGF-β1 stimulation, treatment with low and high TFL concentrations significantly downregulated mRNA expression of activation markers α-SMA (one-way ANOVA, *F*_(3,8)_=7.863 *P* = 0.004), collagen I (one-way ANOVA, *F*_(3,8)_=10.964, *P* = 0.004), and fibronectin (one-way ANOVA, *F*_(3,8)_=11.355, *P* = 0.003) (**Figures 7B–D**). Western blot results also exhibited the same trend (**Figures 8A, B**).

## 4 Discussion

In the current study, we investigated the effect of TFL on liver fibrosis induced by *C. sinensis* in rats. Our results demonstrated that TFL significantly improved liver function in infected rats, as indicated by decreased levels of ALT, AST, and TBA. Pathological examinations revealed that TFL treatment alleviated hepatic swelling, reduced bile duct dilation, and mitigated inflammatory cell infiltration. Furthermore, TFL reduced the number of activated HSCs and downregulated the expression of fibrosis-related markers, including collagen I/III and α-SMA. *In vitro* experiments also confirmed that TFL inhibited the activation of HSCs by decreasing the expression of α-SMA, collagen I, and fibronectin. Collectively, these findings suggest that TFL exhibits potent anti-fibrotic properties against *C. sinensis*-induced liver fibrosis.

The pathogenesis of liver fibrosis is a complex process involving a variety of cytokines and multiple signal transduction pathways. When *C. sinensis* infects humans, the adult worms strongly adhere to the wall of intrahepatic bile ducts through their oral suckers, causing mechanical damage to bile duct epithelial cells. This leads to downregulation of tight junction protein expression and disruption of epithelial barrier integrity (Na et al., 2020). Additionally, excretory-secretory products (ESPs) secreted by the worms activate the TLR4/MyD88/NF-κB signaling pathway, inducing the release of pro-inflammatory cytokines such as IL-6 and TNF-α (Yan et al., 2015). The sustained inflammatory response promotes Th2 immune polarization, with IL-13 upregulating TGF-β1 expression through the STAT6 pathway while inhibiting IFN-γ-mediated fibrosis degradation (Xu et al., 2016). Bile duct obstruction resulting from the infection causes bile stasis, and the accumulated bile acids trigger inflammation by activating the FXR pathway, leading to hepatocyte lipid peroxidation and apoptosis (Li et al., 2017; Panzitt and Wagner, 2021). Furthermore, reactive oxygen species (ROS) generated by the activation of NADPH oxidases (NOX1/4) further promote the activation of HSCs (Jeong et al., 2021). TGF-β1 promotes the phosphorylation of the C-terminal SSXS motif of Smad2/3, facilitating the nuclear translocation of Smad3 and its binding to the COL1A1 promoter region. This results in the upregulation of α-SMA and collagen I expression (Dooley and ten Dijke, 2012). Simultaneously, the continuous high expression of tissue inhibitor of metalloproteinases (TIMP-1) inhibits the activity of matrix metalloproteinases (MMP-1/2), leading to excessive deposition and cross-linking of the extracellular matrix (ECM). Ultimately, these cascading reactions result in periportal fibrosis, the formation of fibrous septa and pseudolobular structures, and the progression to irreversible liver fibrosis or even cirrhosis.

The molecular mechanisms underlying TFL’s inhibition of *C. sinensis*-induced liver fibrosis remain incompletely elucidated, potentially associated with its pharmacological properties of antioxidation, anti-inflammation, and anti-fibrosis. Analysis via liquid chromatography–high resolution mass spectrometry (LC-HRMS) at Tsinghua University’s Pharmaceutical Research Center identified TFL’s top three active components as rutin (36.4%), quercetin (19.8%), and morin (12.5%), collectively constituting two-thirds of its total content. Extensive studies demonstrate these monomeric components exhibit multiple pharmacological activities—including antioxidant, anti-inflammatory, anti-fibrotic, anti-apoptotic, antibacterial, and neuroprotective effects—thus exerting hepatoprotective functions (Rahmani et al., 2023; Pandi et al., 2025).

Regarding antioxidant properties, the three monomeric compounds enhance hepatic antioxidant enzymes (SOD, CAT, GSH-Px), activate the Nrf2/HO-1 signaling pathway, and reduce oxidative products like MDA (Yang et al., 2019; Pandi et al., 2025). Quercetin specifically enhances antioxidant capacity, modulates TLR2/TLR4 and MAPK/NF-κB pathways, suppresses hepatic ROS production, and mitigates CCl_4_-induced oxidative damage (Xu et al., 2019). Rutin demonstrates ROS reduction in cardiac tissue (Enogieru et al., 2018; Rahmani et al., 2023) and elevates hepatic SOD/CAT activity to alleviate oxidative stress in *Schistosoma mansoni* (*S. mansoni*) infections (Hamad, 2023).

For anti-inflammatory effects, the three compounds inhibit hepatic expression of IL-1β, IL-6, TNF-α, and COX-2 while blocking NF-κB activation in cardiac tissue (Rahmani et al., 2023; Pandi et al., 2025). Rutin significantly reduces serum TNF-α, IL-17, and IL-4 in *S. mansoni* infection (Hamad, 2023), aligning with prior studies on TFL’s suppression of *Schistosoma japonicum* (*S. japonicum*)-induced liver fibrosis (Li et al., 2024). Rutin and quercetin additionally inhibit pathogens like *Staphylococcus aureus* and *Escherichia coli* while modulating gut microbiome to indirectly ameliorate inflammation (Qi et al., 2022; Xia et al., 2022; Li et al., 2024). Quercetin also reduces serum ALT/AST/ALP levels and restores TBA in serum/liver (Yang et al., 2019), which is consistent with the current study. Crucially, all three components suppress TGF-β1 expression, a central mediator of fibrosis, providing a mechanistic basis for TFL’s anti-fibrotic efficacy (Pandi et al., 2025).

In terms of anti-apoptotic activity, the three compounds inhibit caspase-3/caspase-9 while upregulating Bcl-2 expression (Rahmani et al., 2023), with rutin reducing splenocyte apoptosis in *S. mansoni* infection (Hamad, 2023). Regarding neuroprotection, rutin operates through multiple pathways: enhancing SOD/CAT/GSH-Px activity and Nrf2/ARE signaling for antioxidation; modulating AGE-NF-κB and TLR4/NF-κB/NLRP3 pathways for anti-inflammation; and regulating PI3K/Akt/mTOR, ERK1/2 signaling, and Bax/Bcl-2 expression for anti-apoptosis (Enogieru et al., 2018; Chunmei and Shuai, 2025). These mechanisms are similar to rutin’s protective actions in other organs.

Collectively, the multifaceted pharmacological activities of TFL’s primary components: targeting oxidative stress, inflammation, fibrosis, apoptosis, and microbial dysregulation, directly counteract key pathological processes in *C. sinensis*-induced fibrogenesis, thereby elucidating its molecular mechanism of action.

As a natural plant extract, TFL possesses numerous advantages. It is derived from litchi seeds, a tropical fruit widely distributed in Southern China, and its safety has been validated through long-term medicinal use. A previous study demonstrated that TFL showed no toxicity in mice even at the maximum oral administration dosage, so it is expected to be safe in larger mammals (Wan et al., 2010). TFL also exhibits no toxicity in parasites: in this study, the parasite burden showed no significant difference between the *Cs*. and the *Cs*. + TFL group. Similarly, in our prior research, adding high concentrations of TFL directly to *S. japonicum* culture media did not affect parasite viability (Li et al., 2024). This indicates that TFL alleviates liver fibrosis not by killing parasites but through antioxidant and anti-inflammatory mechanisms. This is a significant advantage for treating chronic liver fibrosis caused by persistent self-activation of HSCs in *C. sinensis* infection.

Moreover, the anti-fibrotic effects of TFL have been validated across different disease conditions and animal models. This includes chemical-induced liver fibrosis models in rats utilizing agents such as CCl_4_ and dimethylnitrosamine (DMN), as well as authentic pathogen-induced fibrosis models such as *S. japonicum-*induced liver fibrosis in mice (Zhou and Liu, 2015; Yan et al., 2022; Li et al., 2024). Additionally, TFL demonstrates inhibitory activity against HSCs activation within the HSC-T6 cell model. These collective findings indicate that TFL consistently suppresses liver fibrosis across distinct experimental models through a shared core mechanism: specifically, the inhibition of HSCs activation.

Furthermore, while monomeric constituents present in TFL, including rutin, quercetin, and morin, individually exhibit antioxidative, anti-inflammatory, and anti-fibrotic properties, the composite flavonoid mixture comprising TFL incorporates multiple bioactive compounds. This complex composition enables engagement with a broader spectrum of molecular targets to exert its anti-fibrotic effects. Consequently, earlier comparative studies established that individual monomeric components demonstrate inferior efficacy relative to the composite TFL formulation. This evidence highlights the inherent therapeutic advantage of traditional botanical composite preparations over single-component therapeutic agents.

While this study confirmed TFL’s efficacy against *C. sinensis*-induced liver fibrosis, the underlying mechanisms remain incompletely explored. We only established that TFL inhibits HSCs activation by downregulating α-SMA expression. The specific gene targets and pathways through which TFL exerts its anti-oxidative and anti-inflammatory effects to ultimately suppress fibrosis require further investigation. Future research will focus on mechanistic studies, employing network pharmacology, comparative transcriptomics, and proteomics to identify significantly altered genes as potential therapeutic targets. These targets will then be functionally validated using genetic manipulation or protein activators/inhibitors to pinpoint key genes in TFL’s anti-fibrotic actions.

## 5 Conclusions

This study demonstrated that TFL can alleviate *C. sinensis*-induced liver fibrosis, providing experimental support for the development of new anti-liver fibrosis drugs and offering new prospects for the treatment of *C. sinensis* infections.

## Data Availability

The datasets presented in this study can be found in online repositories. The names of the repository/repositories and accession number(s) can be found in the article/**Supplementary Material**.

## Supplementary material

Supplementary Table 1 Mass spectrum of TFL.
